# Evolution of molecular networks underlying plant tissue patterning: insights from conducting tissues

**DOI:** 10.1093/jxb/erag221

**Published:** 2026-05-12

**Authors:** Noel Blanco-Touriñán, Miguel Á Blázquez

**Affiliations:** Instituto de Biología Molecular y Celular de Plantas, Consejo Superior de Investigaciones Científicas—Universitat Politècnica de València, Valencia 46022, Spain; Instituto de Biología Molecular y Celular de Plantas, Consejo Superior de Investigaciones Científicas—Universitat Politècnica de València, Valencia 46022, Spain; University of Manchester, UK

**Keywords:** Bryophytes, conducting cells, land plants, plant evolution, signaling networks, tissue patterning, tracheophytes, vascular tissues

## Abstract

The emergence of land plants involved the progressive elaboration of molecular networks that pattern tissues and define specialized cell types, as illustrated by the evolution of diverse conducting tissues. This review considers the evolution of molecular networks underlying conducting tissues, highlighting how conserved and rewired regulatory modules have generated diverse systems in land plants while providing a framework for studying developmental circuits. While tracheophytes developed complex vascular systems with distinct xylem and phloem, bryophytes evolved functionally analogous cells for water and nutrient transport, including hydroids and leptoids in mosses and pegged rhizoids in complex thalloid liverworts. Fossil evidence, such as the early Devonian plant *Horneophyton*, suggests that multifunctional conducting cells might represent early forms of conducting cells that pre-date the divergence of modern tracheophyte vascular tissues and bryophyte conducting cells, indicating that key components of their developmental machinery were already present in early land plants. Across plant lineages, conducting tissues have evolved through the redeployment of shared genetic modules, lineage-specific innovations, and rewiring of existing networks, shaping diverse patterns of tissue differentiation. A striking example of this divergence is found in certain liverworts, where water-conducting cells have been associated with pegged rhizoids and appear to be controlled by independent developmental mechanisms. A comparative approach is thus essential to understand how these crucial tissues emerged and diversified over millions of years of plant evolution, while also providing a framework for investigating the evolution of other developmental circuits.

## Teaser

This review explores molecular network evolution underlying conducting tissues, highlighting how conserved and rewired regulatory modules generated diverse systems in land plants while providing a framework for studying developmental circuits.

## Introduction

**Fig. 1.**
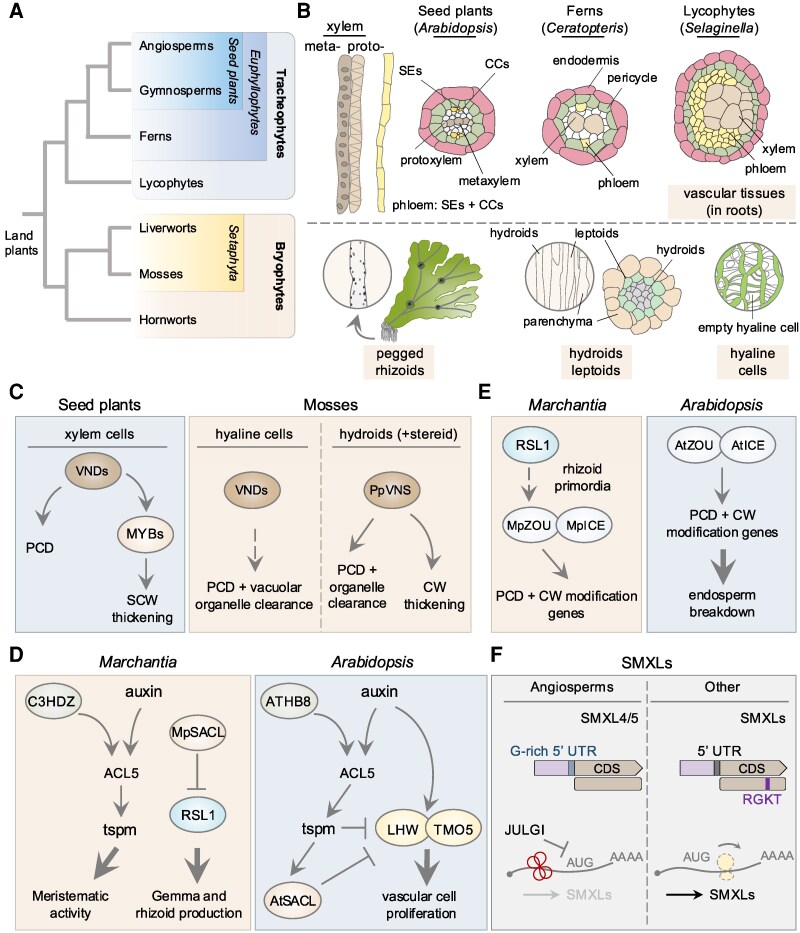
Comparison of conducting-cell types and regulatory modules in land plants. (A) Phylogenetic tree illustrating the major extant land plant groups. (B) Comparative overview of conducting structures across representative lineages, highlighting the diversity of water- and food-conducting cell types in bryophytes (e.g. hydroids, leptoids, pegged rhizoids) versus tracheophytes (true vascular tissues). True vascular conducting tissues include phloem, comprising sieve elements (SEs) and companion cells (CCs), and xylem, which includes protoxylem and metaxylem characterized by spiral/annular and pitted/reticulate secondary wall thickenings, respectively. Root cross-sections for Arabidopsis, *Ceratopteris*, and *Selaginella* are shown. Certain liverworts contain pegged rhizoids, dead cells with internal wall thickenings (‘pegs’) that facilitate water movement but do not form continuous strands. In mosses, hydroids are elongated, dead cells that transport water along internal strands of the gametophyte. Leptoids are living cells responsible for sugar and nutrient transport. Parenchyma cells provide structural support. Hyaline cells in *Sphagnum* are also shown; although they do not act as genuine conducting cells, they might form by mechanisms similar to some water-conducting cells (see text). (C–F) Illustrative examples of major evolutionary patterns identified from comparative analyses. (C) Repeated recruitment of the same ancestral module in multiple lineages, as exemplified by VND transcription factors that govern secondary cell wall deposition and programmed cell death in both bryophytes and tracheophytes. (D, E) Lineage-specific innovation via rewiring of ancestral modules, exemplified by ZOU–ICE1 in liverworts (*Marchantia polymorpha*) and SACL–ACL5 in tracheophytes (Arabidopsis). (F) Pathway rewiring and the emergence of novel regulatory modules within a lineage, illustrated by the JULGI–SMXL module in angiosperms. This module regulates phloem development independently of strigolactone signaling due to loss of the strigolactone-responsive RGKT domain and acquisition of a G-rich 5′-UTR that serves as the JULGI targeting site. PCD, programmed cell death; (S)CW, (secondary) cell wall; tspm, thermospermine.

The establishment of spatially organized tissues in land plants relies on gene regulatory networks that control cell identity, differentiation, and patterning. Traditionally, vascular tissues have provided a paradigmatic framework to study these processes, distinguishing tracheophytes from their non-vascular relatives (Fig. 1A, B). The vascular tissues include xylem, which conducts water and provides mechanical support for life on land, and phloem, which transports sugars and other signaling molecules throughout the plant (Lucas *et al*., 2013). Xylem is composed of elongated cells that develop secondary cell walls (SCWs) impregnated with lignin and undergo programmed cell death (PCD), creating rigid, hollow conduits for long-distance water transport (Lucas *et al*., 2013; Růžička *et al*., 2015). The pits of xylem cells allow for lateral movement of water between tracheids, while in vessel elements (angiosperms), perforations also connect cells directly, allowing faster water flow. In contrast, phloem consists of living cells, including sieve elements and companion cells, that transport sugars and signaling molecules while maintaining symplastic continuity (Hardtke, 2023). These vascular cell types are established during primary growth, when vascular tissues differentiate from procambial cells derived from primary meristems and form discrete xylem and phloem strands that support longitudinal organ growth. In many plants, including the model species Arabidopsis, vascular tissues develop further during secondary growth, a process driven by the activity of lateral meristems, particularly the vascular cambium. The cambium generates secondary xylem toward the inside and secondary phloem toward the outside, enabling radial expansion of stems and roots, and enhancing both mechanical support and hydraulic capacity (Wang *et al*., 2023).

Some bryophytes have evolved specialized water-conducting cells (WCCs) and food-conducting cells (FCCs) for efficient transport of water and nutrients along the gametophyte (Brodribb *et al*., 2020). In mosses, hydroids are the main WCCs, consisting of elongated, dead cells that transport water along internal strands (Fig. 1B). They can be either perforate or imperforate but, unlike tracheophyte tracheids, they lack lignified SCWs (Lucas *et al*., 2013). Functionally, hydroids are analogous to tracheids, whereas leptoids are the FCCs and resemble sieve elements. In liverworts, pegged rhizoids are dead-at-maturity cells that transport water. They are termed ‘pegged’ because their cell walls contain small, internal wall thickenings (‘pegs’). While earlier studies proposed that these structures reinforce the cell wall against collapse and/or increase surface area for absorption, more recent time-lapse imaging provides support for a role in water transport. Such experiments have shown that passive solute flow into pegged rhizoids occurs following PCD, when membrane integrity is lost (Lu *et al*., 2024). Unlike moss hydroids, pegged rhizoids do not form continuous internal strands and are often described as externally positioned epidermal derivatives, leading to debate over whether they represent true WCCs or a functional analog. However, they can also occur within internal regions of the thallus, for example within the carpocephalum stalk. In these tissues, transport experiments have demonstrated their involvement in short-distance water conduction and revealed functional similarities to both hydroids and tracheary elements (Duckett *et al*., 2014). Together with hydroids and leptoids, pegged rhizoids illustrate the diverse strategies by which bryophytes achieve transport in the absence of a true vascular system. Alternative strategies also occur within liverworts. For example, the genus *Haplomitrium* lacks pegged rhizoids and instead possesses internal conducting cells with distinct cell wall compositions (Ligrone *et al*., 2000). Besides conducting cells, some mosses also possess hyaline cells that act primarily as water-storage compartments rather than true conducting cells. Although they do not appear to contribute directly to water transport, they are included here because several aspects of their differentiation resemble those of vessel elements, making them relevant for comparisons of regulatory pathways (Kremer and Drinnan, 2004).

While previous influential reviews have thoroughly covered physiological, anatomical, palaeobotanical, and environmental aspects of conducting cells (Lucas *et al*., 2013; Woudenberg *et al*., 2022; Hetherington, 2024; Agustí and Blanco-Touriñán, 2025), their evolutionary history as inferred from molecular developmental mechanisms has been discussed to a much lesser extent. This largely reflects the historical concentration of molecular data in angiosperms, with comparatively little functional insight from other plant lineages. However, recent advances in genetics, comparative genomics, and functional studies across diverse taxa are now beginning to overcome this limitation (Box 1). These emerging data have identified foundational genes conserved across vascular plant lineages, traced the evolutionary history of their regulatory modules, and, in parallel, proposed the first molecular mechanisms controlling conducting tissue formation in liverworts. These molecular insights indicate that the development of conducting cells involved both independent innovations and convergent recruitment of similar/conserved regulatory modules between major plant lineages. Here, we focus on these molecular regulators, summarizing their roles across plant groups and using comparative analysis to discuss their evolution. Understanding these networks is key to understanding how vascular and analogous conducting tissues emerged and diversified over millions of years of evolution.

Box 1. Key developments in understanding the evolution and molecular regulation of conducting tissues in land plants.

The following studies advanced our understanding of how vascular development is orchestrated through both conserved and lineage-specific regulatory pathways. Comparative and genetic analyses have started to reveal how these regulators diversified, gained new functions, and integrated within ancestral and newly evolved regulatory circuits. An overview of some of these and other studies is shown in Fig. 1.

- Xiao *et al*. (2026) showed that in the lycophyte *Selaginella moellendorffii*, auxin and cytokinin regulate vascular development via a clear task separation, with auxin controlling cell proliferation and cytokinin controlling cell differentiation. Using cross-species transcriptomics, the authors found that the AUXIN/INDOLE-ACETIC ACID (AUX/IAA) and CYTOKININ OXIDASE (CKX) proteins are functionally conserved, but their regulatory circuits diverged compared with euphyllophytes. This study provides a striking example of evolutionary rewiring in hormonal regulation of vascular tissues while maintaining conserved protein functions.
- Xue *et al*. (2025) built a unified single-cell atlas of shoot apices from six vascular plants spanning major evolutionary groups (i.e. angiosperms, gymnosperms, ferns, and lycophytes) and performed comparative analyses to identify foundational vascular genes. This study also revealed that anatomically identified Strasburger (albuminous) cells in ferns and gymnosperms possess transcriptional signatures similar to those of companion cells.
- Park *et al*. (2025) showed that the RNA-binding protein JULGI represses *SMXL4/5* expression in angiosperms by binding G-rich elements in the 5′-UTR of SMXL4/5. Because angiosperm SMXL4/5 proteins have lost the RGKT motif required for strigolactone sensitivity, this repression decouples phloem development from strigolactone-mediated control of lateral branching. In gymnosperms and seedless vascular plants, SMXL4/5 homologs remain strigolactone-sensitive, indicating that this JULGI–SMXL4/5 regulatory module is an angiosperm-specific innovation.
- Solé-Gil *et al*. (2025) studied several modules (including auxin, C3HDZ, ACL5, SACL, and LHW–TMO5) in *Marchantia polymorpha*, revealing how thermospermine-dependent circuit elements followed divergent evolutionary trajectories in bryophytes and tracheophytes, ultimately shaping lineage-specific developmental processes.
- Lu *et al*. (2024) showed that the water-conducting tissue in *M. polymorpha* (i.e. the pegged rhizoids) differentiates via a ZHOUPI/ICE-dependent programmed cell death pathway. This process is independent of *VND* genes, highlighting that pegged rhizoids evolved separately from other water-conducting cells.

## Vascular tissues in tracheophytes

Tracheophytes, a monophyletic group including lycophytes and euphyllophytes (ferns and seed plants; Fig. 1A), share a branched sporophyte-dominated life cycle and vascular tissues, with tracheary elements forming xylem for water and nutrient transport, and sieve elements forming phloem for photoassimilate transport (Fig. 1B; Simpson, 2010). These traits were traditionally traced back to the earliest vascular plants, the Devonian rhyniophytes, which were assumed to possess a primitive protostele in which a few layers of phloem surrounded the xylem composed of tracheids with annular or spiral secondary wall thickenings (Satterthwait and Schopf, 1972; Edwards, 2003; Kerp, 2018; Hetherington and Dolan, 2019). However, recent high-resolution analyses of *Horneophyton lignieri* reveal that these early land plants lacked distinct xylem and phloem, instead having transfer-cell-like tissues (Kenrick and Long, 2026). This suggests that the ancestral vascular system might have been composed of a single type of conducting tissue capable of both water and solute transport. Within tracheophytes, the progressive increase in sporophyte height was accompanied by the evolution of stiffer vascular tissue that provides both mechanical support and efficient water transport. During this process, gradual modifications in the arrangement of xylem cells enhanced drought resistance by reducing hydraulic failure, that is, preventing interruptions in water flow (Bouda *et al*., 2022).

### Seed plants

Tracheophyte roots consistently show a protostele organization, characterized by a single central strand of vascular tissues, whereas their shoot vasculature is extremely diverse in its structure (Decombeix *et al*., 2019). Seed plants (also called spermatophytes) include gymnosperms and angiosperms, and their shoots display a derived eustele composed of discrete vascular bundles, each containing both xylem and phloem. Research in Arabidopsis has greatly advanced our understanding of (pro)cambium maintenance and xylem/phloem differentiation (for comprehensive reviews see Hardtke, 2023; Wang *et al*., 2023; Kondo and Ohashi-Ito, 2025). Although the extent to which these regulatory mechanisms are conserved across tracheophytes or across different organs remains unclear (Blanco-Touriñán and Hardtke, 2023), comparative analyses across vascular plant lineages suggest a core set of conserved (or ‘foundational’) vascular genes (Xue *et al*., 2025). These genes are defined as deeply conserved across vascular plants and expressed in ways that support the functional identity of key vascular cell types, such as xylem and phloem. Representative examples of these conserved vascular genes include the class III HD-ZIP (C3HDZ) transcription factor (TF) genes encoding PHABULOSA (PHB) and CORONA (ATHB-15), as well as TARGET OF MONOPTEROS 5 (TMO5), VASCULAR-RELATED NAC-DOMAIN 1 (VND1), XYLEM CYSTEINE PEPTIDASE 1 and 2 (XCP1, XCP2), ACAULIS 5 (ACL5), and PHLOEM INTERCALATED WITH XYLEM (PXY), and the auxin influx carrier genes encoding AUXIN1 (AUX1) and LIKE-AUX1 1 (LAX1) for xylem, and ALTERED PHLOEM DEVELOPMENT (APL), SMAX1-LIKE 5 (SMXL5), PHLOEM EARLY DOF (PEAR), and the receptor-like kinases BARELY ANY MERISTEM 1 and 3 (BAM1, BAM3) for phloem. This section focuses on a subset of these genes and their regulatory networks in Arabidopsis, the most studied angiosperm, as well as on additional genes that have been studied in other plant species to understand their conservation.

During primary vascular development in the diarch root of Arabidopsis, the xylem axis is established with central metaxylem cells flanked by protoxylem at the periphery, while the two phloem poles are positioned lateral to the procambial domains (Fig. 1B). Key regulatory modules orchestrate this patterning. An auxin response maximum along the xylem axis promotes the transcription of the cytokinin (CK) signaling inhibitor *ARABIDOPSIS HISTIDINE PHOSPHOTRANSFER PROTEIN 6* (*AHP6*), promoting protoxylem formation at the periphery. In contrast, central xylem cells express C3HDZ TFs that suppress *AHP6*, preventing protoxylem formation and specifying metaxylem identity (Carlsbecker *et al*., 2010; Bishopp *et al*., 2011). High CK signaling in the adjacent procambial cells promotes the expression and lateral localization of PINFORMED (PIN) auxin efflux carriers, reinforcing auxin accumulation in xylem cell files (Bishopp *et al*., 2011). The LONESOME HIGHWAY (LHW)–TMO5 complex promotes xylem cell divisions via CK, a process under the control of an incoherent feed-forward loop where auxin and the C3HDZ TF ATHB8 induce thermospermine production via ACAULIS5 (ACL5) (Fig. 1D; Baima *et al*., 2014). Thermospermine limits this proliferation by repressing the translation of LHW and promoting that of SAC51-LIKE (SACL) bHLH TFs. These SACL TFs compete with TMO5 for LHW heterodimerization, which ultimately restricts CK production and new xylem cell formation (Katayama *et al*., 2015; Vera-Sirera *et al*., 2015; Ko *et al*., 2026). Xylem differentiation is then mediated by VASCULAR-RELATED NAC (NAM, ATAF1/2, CUC2) TFs, which direct SCW formation and PCD (Kubo *et al*., 2005; Lehmann and Schneider, 2025).

Primary phloem is formed by sieve elements (SEs) and companion cells (CCs) (Fig. 1B). During differentiation, SEs lose most organelles to form connected sieve tubes and are metabolically supported by CCs through plasmodesmata (Hardtke, 2023). SHORT-ROOT (SHR) promotes cell divisions, while phloem differentiation is controlled by a genetic network that includes both positive (e.g. PEARs, APL) and negative regulators [e.g. the CLAVATA3/EMBRYO SURROUNDING REGION RELATED (CLE) peptides and their BAM3 receptor] (Helariutta *et al*., 2000; Hardtke, 2023).

Meanwhile, during secondary growth high auxin signaling defines the stem-cell organizer of vascular cambium in Arabidopsis, and this auxin maximum is modulated by gibberellins (Smetana *et al*., 2019; Mäkilä *et al*., 2023). CLE peptide-receptor pairs modulate the activity of these cambial stem cells and the differentiation of their derivatives. *CLE41* and *CLE44* encode the TRACHEARY ELEMENT DIFFERENTIATION INHIBITORY FACTOR (TDIF), a peptide secreted by phloem and perceived by the PXY receptor in hypocotyl cambial cells. TDIF–PXY signaling promotes cambial cell divisions through WUSCHEL-HOMEOBOX RELATED 4 (WOX4) factors and inhibits xylem differentiation (Etchells *et al*., 2013). Similar patterns are observed in *Populus* stems, where peak auxin levels coincide with actively dividing cambial cells (Immanen *et al*., 2016; Liu *et al*., 2025). These findings suggest that regulatory networks controlling cambial activity are conserved across angiosperms, a conclusion further supported by the identification of common TFs, including WOX4 and several C3HDZ TFs, as key regulators of cambial activity (Du *et al*., 2011; Robischon *et al*., 2011; Zhu *et al*., 2013, 2018; Kucukoglu *et al*., 2017) and by comparative single-nucleus RNA sequencing of *Populus* and Arabidopsis (Conde *et al*., 2022).

In gymnosperms, much less is known about the regulatory mechanisms underlying vascular development. More than a century ago, Eduard Strasburger described the occurrence of albuminous cells (also known as Strasburger cells) in the phloem of gymnosperms (Strasburger, 1891; Volkmann *et al*., 2012). These cells are involved in phloem loading and might be functionally analogous to companion cells in angiosperms, a hypothesis supported by their similar transcriptional profiles as revealed by recent single-cell analyses (Xue *et al*., 2025). Beyond phloem organization, such analyses in *Cunninghamia lanceolata* have also revealed previously unrecognized cellular heterogeneity within gymnosperm xylem, including a cell population not described in angiosperms (Shuai *et al*., 2025).

In summary, studies in angiosperms (particularly Arabidopsis and *Populus*) have revealed detailed regulatory frameworks governing primary and secondary vascular development, whereas our knowledge in gymnosperms remains relatively poor. Nonetheless, recent comparative studies indicate that many key regulators are deeply conserved across tracheophytes. Extending functional and mechanistic analyses beyond angiosperm models will therefore be essential for understanding how conserved genetic programs have been redeployed to generate the remarkable diversity of vascular architectures observed in seed plants.

### Ferns

As the sister group to seed plants (Fig. 1A), ferns occupy a key phylogenetic position. Their sporophytes form true vascular tissues, yet their free-living gametophytes develop rhizoids, resembling bryophytes (Conway and Di Stilio, 2020). In leaves (fronds), ferns exhibit considerable diversity in vascular architecture, which might partly arise from developmental interactions that couple organ patterning with stem vasculature (Suissa, 2025). In roots, cellular-level analyses of the model fern *Ceratopteris* have revealed a cylindrical core of xylem flanked by phloem poles (Fig. 1B; Hou and Hill, 2004; Aragón-Raygoza *et al*., 2020). This root arrangement resembles the radial vascular patterning observed in roots of angiosperms such as Arabidopsis, suggesting that key aspects of vascular tissue specification might have arisen early in euphyllophyte evolution. Taken together, the diversity observed in shoot vasculature and the conserved root pattern suggest that some aspects of vascular differentiation might be regulated by conserved mechanisms, while others could be lineage- or organ-specific.

Emerging evidence indicates that ferns employ conserved signaling pathways to control vascular differentiation, such as CLE peptide- and auxin-mediated mechanisms. For example, CLE peptides might regulate xylem development as treatment with TDIF inhibits the differentiation of procambial cells into xylem in the fern *Adiantum aethiopicum* (Hirakawa and Bowman, 2015). Similarly, auxin transport influences vascular patterning during sporophytic development in ferns (Woudenberg *et al*., 2024). Although fern fronds evolved independently and are not homologous to angiosperm leaves, inhibition of auxin transport in *Ceratopteris richardii* results in fronds with altered vascular patterns, characterized by higher vein density and connectivity, suggesting that efficient auxin transport normally restricts excessive vein formation and interconnection. Moreover, blocking auxin transport in gametophytes promotes cellular reprogramming and the ectopic initiation of sporophyte development. Under these conditions, root-like structures can arise from gametophytic tissues and develop vascular tissues with spiraling secondary wall thickenings (Woudenberg *et al*., 2024). More direct evidence for hormonal regulation of vascular differentiation in ferns comes from other species (Ma and Steeves, 1992). For instance, in the aquatic fern *Azolla filiculoides* both auxin and CK promote xylem differentiation toward the root tip and modulate SCW deposition patterns (De Vries *et al*., 2016), and it has been proposed that these effects might involve hormonal crosstalk (Xiao *et al*., 2026).

Further understanding of the genetic regulation of vascular development in ferns has been established through studies identifying TFs that probably interact with these hormonal pathways. For example, in *C. richardii* the *WOX* TF gene *WUS* is expressed during early root development and in the vasculature; RNAi-mediated knockdown of *WUS* significantly compromises root formation and leads to a modest reduction in the number of cells that can be interpreted as phloem in anatomic analyses (Youngstrom *et al*., 2022). Vascular *WOX* genes in seed plants also tend to show relatively subtle phenotypes. In Arabidopsis, *WOX4* and *WOX14* act redundantly in the regulation of vascular cell division, while vascular organization remains largely unaffected in the loss-of-function mutants, suggesting that these genes primarily regulate vascular cell proliferation rather than vascular patterning. Similarly, the phenotype observed after knockdown of *WUS* in *C. richardii* might reflect a role in regulating vascular cell proliferation or maintenance, potentially acting downstream of the complex auxin and CK signaling described above. Taken together, the available evidence suggests that ferns utilize conserved hormonal and transcriptional regulators to control vascular differentiation. However, the scarcity of functional genetic studies currently limits our understanding of how these regulatory networks were assembled during euphyllophyte evolution.

### Lycophytes

Lycophytes represent one of the earliest-diverging vascular plant lineages and exhibit striking diversity in growth patterns, ranging from the zig-zagging shoots of *Selaginella kraussiana* to the frond-like branches of *S. moellendorffii* (Spencer *et al*., 2021). A distinctive feature of lycophytes is the microphyll, a simple leaf with a single, unbranched vein. Their stems typically contain a central protostele (Spencer *et al*., 2021), although in some *Selaginella* species, such as *S. kraussiana*, the stem contains two separate vascular strands, each consisting of xylem surrounded by phloem (Floyd and Bowman, 2006). Their roots branch dichotomously, and their stele is organized into one xylem strand opposite a phloem strand (Fig. 1B; Yang *et al*., 2023). This apparent morphological simplicity provides a useful context for studying the gradual evolution of vascular tissues.

Recent evidence suggests that auxin promotes vascular cell proliferation in *S. kraussiana* roots whereas cytokinin drives cell differentiation, revealing a clear task separation between these two hormones (Xiao *et al*., 2026). Moreover, polar auxin transport (PAT) mediated by PIN-FORMED homologs coordinates vascular patterning in both stems and leaves. Pharmacological inhibition of PAT produces thicker veins composed of numerous, wider, and disorganized tracheids that fail to extend to the leaf tip (Sanders and Langdale, 2013). Application of the auxin analog 1-naphthaleneacetic acid has no effect, confirming that it is disrupted auxin transport rather than auxin level *per se* that causes the phenotype. Comparable enlargement of the stem vasculature suggests that PAT broadly coordinates vascular patterning across organs (Sanders and Langdale, 2013). This mechanism probably depends on the PIN homologs *PINR* and *PINS*, which are highly expressed in the vasculature and are thought to coordinate long-range auxin transport between organs in *S. kraussiana* (Spencer *et al*., 2023). Consistent with a conserved role of canonical PIN proteins in *Selaginella*, heterologous expression of the *S. moellendorffii* PIN *PINR* rescues the shoot/root patterning defects of the Arabidopsis *pin1/3/4/7* mutant (Zhang *et al*., 2020). Complementary insights into vascular patterning in *S. kraussiana* come from analyses of expression of C3HDZ genes. *C3HDZ2* is strongly expressed in the apical cells and early provascular strands of the shoot apex, marking the position of the future two separate vascular strands (meristeles) even before vascular tissues become anatomically evident, and later becomes confined to the first-maturing tracheary elements in both stem and microphyll (Floyd and Bowman, 2006). By contrast, *C3HDZ1* is expressed in the developing microphyll and in the outer cell layers of the stem provascular strands that differentiate into phloem and pericycle.

To date, the lack of an efficient and reproducible genetic transformation protocol for *Selaginella* species has limited functional studies of vascular development (Fang *et al*., 2021). However, the availability of genome sequences coupled with transcriptomic and heterologous complementation experiments has shed new light on the cellular and molecular bases of vascular tissue development in this lineage (Banks *et al*., 2011; Ferrari *et al*., 2020; Liu *et al*., 2023; Yang *et al*., 2023). In particular, studies of *S. moellendorffii* roots have shown that dichotomous root branching can be reconstructed through an *in vitro* developmental time-course, in which repetitive apex bifurcations are used to capture successive stages of root meristem duplication. These data are consistent with the idea that dichotomous branching might originate from a symmetric division of the initial cell (IC), which produces two new ICs that give rise to twin root meristems (Motte *et al*., 2022). Transcriptomic time-course analyses of root branching have revealed transient changes in gene expression associated with meristem duplication and cell division activity. Genes related to vascular development are dynamically expressed during this process, including *LHW* and *TMO5*, which may play roles in early vascular development (Motte *et al*., 2022). Heterologous studies show that *SmLHW* and *SmTMO5* can complement the respective Arabidopsis *lhw* and *tmo5* mutant phenotypes, supporting functional conservation across vascular plants (Lu *et al*., 2020). Spatial transcriptomics has further shown that *TMO5* and *VND1* are expressed in the xylem in *S. moellendorffii* and show strong co-expression in root tissues, whereas *VND2* is expressed in phloem, indicating tissue-specific expression among these genes (Yang *et al*., 2023). For comparison, in Arabidopsis *VND1* and *VND2* are expressed in immature xylem cells within the meristem (Lehmann and Schneider, 2025). This suggests partial conservation of VND roles in xylem development across vascular plants, accompanied by lineage-specific differences in their spatial deployment. Single-cell RNA sequencing of *S. kraussiana* root tips has also revealed putative vascular cell clusters expressing the GRAS family gene *SHORT-ROOTa* (*SHRa*) and peptide signaling pathway genes such as *CLE-RESISTANT RECEPTOR KINASE* (*CLERK*) and *BARELY ANY MERISTEM 1* (*BAM1*) (Liu *et al*., 2023). Heterologous expression of *SkSHRa* can rescue Arabidopsis *shr* mutant phenotypes, indicating that its molecular function in regulating cell identity and vascular development is conserved across vascular plants (Yang *et al*., 2023). In summary, studies using both classical and advanced technologies have mapped expression patterns of key molecular players in lycophyte vascular development, and their functional conservation has been tested through heterologous complementation; however, functional studies remain a major gap.

## Conducting cells in bryophytes

A contemporary view of vasculature evolution posits that cells specialized in water and photoassimilate transport evolved independently from relatively simple parenchymatous conducting cells present in the last common ancestor of land plants. These ancestral conducting elements diversified along different evolutionary trajectories in tracheophytes and bryophytes, giving rise to analogous (but not homologous) cell types (Woudenberg *et al*., 2022). In bryophytes, several types of water-conducting cells have been described, including hydroids in several moss and liverwort clades and pegged rhizoids in certain liverworts (Fig. 1B). In addition, FCCs named leptoids and leptoid-like have been found in certain mosses and liverworts, respectively, although they might also contribute to water transport (Ligrone *et al*., 2000; Okafor and Budke, 2024). Hydroids and leptoids are usually associated with stereids in mosses, which are elongated, supportive cells that are not lignified, but their cell walls are enriched in (1-4)-ß-D-galactans (Chernova *et al*., 2024).

Genes specifically involved in the regulation of leptoid development have not been identified yet. However, ultrastructural and biochemical analyses suggest that leptoid development involves callose-mediated wall specialization, paralleling processes observed in sieve elements. Indeed, callose synthases have been identified and are expressed in the moss genus *Polytrichum*, indicating a conserved role in cell wall modulation during the maturation of conducting cells (Renzaglia *et al*., 2024). Similarly, leptoid function appears to rely on conserved sugar transport mechanisms: homologs of SWEET (sugar efflux) and SUT (sucrose–H^+^ symporter) transporters are expressed in stem-like (caulid) and leaf-like (phyllid) conducting tissues of the moss *Physcomitrium patens*, suggesting recruitment of ancient carbohydrate transport modules for photoassimilate movement (De Vries *et al*., 2018; Fürst-Jansen *et al*., 2022).

Recent molecular studies have revealed that bryophyte WCCs are patterned and differentiated by regulatory pathways partially conserved with those of tracheophyte vascular tissues. The midrib, the central strand of many moss gametophores, is a key anatomical feature where hydroids and associated support cells predominate. Its specification and organization are controlled by several developmental regulators. In *P. patens*, suppression of class III HD-ZIP expression leads to defects in midrib formation (Yip *et al*., 2016) while *LATERAL SUPPRESSOR 1* (*LAS1*) and *LAS2* promote formative cell divisions that establish the midrib and the alignment of central conducting cells. Loss of *LAS1/2* function results in disorganized or reduced midribs and altered cell division planes (Ge *et al*., 2022; Horiuchi *et al*., 2025). Similarly, *SHR* contributes to the spatial organization and polarity of the central strand, coordinating periclinal and anticlinal divisions necessary for WCC file formation in *P. patens* (Ishikawa *et al*., 2023). These findings indicate that the establishment of conducting domains in bryophytes and tracheophytes shares deep genetic ancestry.

Within the developing hydroid strand, VND TFs act as master regulators of differentiation. In *P. patens*, *VND* genes regulate both the deposition of specialized secondary walls and the PCD that transforms living precursors into functional, dead hydroids (Xu *et al*., 2014). A related role for VND factors has been suggested in *Sphagnum*, where they might control vacuole-mediated death signaling during the formation of hyaline cells, the large, empty water-storage cells characteristic of peat moss leaves (Fig. 1B) (Liu *et al*., 2024).

In liverworts, water conduction can also occur through pegged rhizoids, which clearly evolved independently from other WCCs and are controlled by distinct regulatory modules. In *Marchantia polymorpha*, the ZHOUPI (ZOU) and INDUCER OF CBF EXPRESSION 1 (ICE1) bHLH TFs were recently shown to promote the formation of pegged rhizoids by regulating cell differentiation and wall invagination processes (Lu *et al*., 2024). This ZOU–ICE1 module generates specialized epidermal cells for water transport, demonstrating an independent genetic mechanism from the VND-mediated pathway (as the VND ortholog NAC5 is not involved).

Taken together, these findings indicate that bryophytes possess a diverse array of conducting cell types that rely on both ancestral cellular features and lineage-specific innovations. In the next section, we will focus on comparative analyses and propose several patterns in the regulation of plant conducting tissues.

## Lessons on conducting-tissue evolution from comparative analyses

Unlike the largely conserved water-homeostasis mechanisms in plants (Zhu *et al*., 2025), comparative analyses reveal that conducting cells evolved via distinct strategies in different lineages, involving the repurposing of an ancestral genetic toolkit together with developing lineage-specific innovations. In a few cases, these innovations have resulted in the convergent co-option of similar genetic networks. The existence of an ancestral toolkit in land plants is supported by the presence of orthologs in bryophytes for many genes implicated in vascular development in tracheophytes (Bowman *et al*., 2017), although several key vascular regulators are absent. Analysis of these orthologs reveals three main patterns (Fig. 1C–F). First, some ancestral genes or modules have been repeatedly recruited for different cellular strategies, either due to conservation or convergent co-option (e.g. VNDs). Second, ancestral modules that contribute to (water-) conducting cell differentiation have been differentially recruited and rewired between bryophytes and tracheophytes, resulting in distinct developmental roles and regulatory relationships (e.g. ZOU–ICE1, LASs, TMO5–LHW, SACL–ACL5) (Lu *et al*., 2020, 2024; Solé-Gil *et al*., 2025). Third, additional lineage-specific innovations and pathway rewiring have diversified these programs within particular clades, generating new regulatory modules or cellular strategies. These three patterns are discussed in more detail below.

The first pattern of repeated recruitment of ancestral genes for diverse cellular strategies is exemplified by VND-like TFs (Fig. 1C). For instance, VND factors control hydroid differentiation in the bryophyte *Physcomitrium patens* by triggering secondary wall deposition and PCD (Ge *et al*., 2022). This mirrors the role of VNDs in tracheophyte xylem differentiation, showing the redeployment of this module within simpler cellular contexts (Lehmann and Schneider, 2025). More broadly, other TF families also show repeated involvement in cell wall differentiation programs across land plants. For example, *KNOX* genes influence SCW growth in both *Marchantia* and Arabidopsis (Etchells *et al*., 2013; Dierschke *et al*., 2024). It has been hypothesized that such ancient regulatory networks controlling cell wall modification might have originally functioned in other tissues, such as the sporangium, and were later combined with pre-existing WCC types during tracheophyte evolution, ultimately contributing to the emergence of tracheids (Dierschke *et al*., 2024). However, while VND-like modules show a repeated association with conducting cell differentiation across land plants—consistent with either conservation or recurrent co-option—the extent to which *KNOX* genes participate in such programs remains unresolved due to the lack of comparative studies across different lineages and conducting cell types.

Beyond the repeated redeployment of the same ancestral genes, the second pattern highlights how different ancestral modules have been differentially recruited and/or rewired in bryophytes and tracheophytes, generating distinct cellular strategies across these lineages (Fig. 1D). For example, the heterodimeric TMO5–LHW TF complex is rate-limiting for vascular cell proliferation in Arabidopsis. TMO5 acquired the ability to dimerize with LHW at the origin of land plants, while a second innovation in LHW coincided with the emergence of tracheophytes, resulting in its obligate heterodimerization with TMO5, thereby establishing a critical function in vascular development (Lu *et al*., 2020). Another example is the acquisition of a novel upstream open reading frame in the ancestral tracheophyte *SACL* mRNA, which enabled ACL5-regulated vascular functions, whereas *ACL5* and *SACL* in *Marchantia polymorpha* control separate developmental processes unrelated to water transport (Solé-Gil *et al*., 2025). Similarly, in the moss *Physcomitrium patens*, *LAS1* and *LAS2* promote formative divisions that establish the midrib and align central conducting cells, a role distinct from *LAS* function in tracheophytes, providing another clear example of lineage-specific regulatory rewiring (Ge *et al*., 2022). Analogous lineage-specific recruitment during water-conducting cell development can also be seen with *M. polymorpha* ZOU and ICE1 (Fig. 1E). In contrast to the widespread use of VND-like regulators across most lineages in conducting cell differentiation, *Marchantia* appears to employ a distinct regulatory module that is not typically associated with water transport. Consistent with this, orthologous factors in flowering plants function in the endosperm, where they control cell wall remodeling and PCD (Denay *et al*., 2014; Doll *et al*., 2023), indicating that this module has been co-opted into multiple, functionally distinct developmental contexts. Given that its role in conducting cell differentiation appears to be restricted to liverworts, this might represent a lineage-specific innovation rather than a broadly conserved component of the ancestral toolkit.

These patterns of redeployment and divergence are not limited to comparisons between bryophytes and tracheophytes. Even within a single lineage such as tracheophytes, vascular development shows both molecular and cellular diversity, arising through lineage-specific innovations and pathway rewiring (the third pattern), including functional divergence or loss of regulatory networks. Lycophytes such as *S. moellendorffii* show less complexity in gene structure, alternative splicing, and regulatory networks governing vascular development (Zhu *et al*., 2017). For example, hormonal control of vascular development appears to have been rewired in this lineage: whereas auxin–cytokinin crosstalk coordinates both vascular cell proliferation and differentiation in several euphyllophytes, including angiosperms such as Arabidopsis and *Triticum* as well as ferns such as *Azolla*, *S. moellendorffii* shows a clear task separation in which auxin primarily promotes cell proliferation while cytokinin specifically triggers cell differentiation (Xiao *et al*., 2026). Moreover, the CLE-family peptide TDIF inhibits xylem differentiation in Arabidopsis and other euphyllophytes such as *Ginkgo biloba* (a gymnosperm) and *Adiantum aethiopicum* (a fern), but not in lycophytes such as *S. kraussiana* (Hirakawa and Bowman, 2015). Within fern lineages, phylogenetic analyses of genes involved in cell wall biosynthesis suggest that their cells have probably evolved cell wall structures with properties distinct from those of flowering plants (Ali *et al*., 2025). These cases are consistent with the existence of a common ancestral module for vascular differentiation, followed by lineage-specific loss or functional divergence. This is also compatible with the idea that euphyllophytes evolved additional layers of peptide-based control, such as the TDIF–PXY–WOX4 module that coordinates (pro)cambial activity and xylem differentiation.

Further refinement of regulatory circuits is evident in seed plants (Fig. 1F). For instance, the RNA-binding protein JULGI and ancestral SMXLs are co-expressed in phloem from lycophytes to angiosperms. In angiosperms, however, SMXL4/5 proteins differ in two key ways: they lack the RGKT motif that targets other SMXLs for strigolactone-mediated degradation, thereby decoupling phloem development from strigolactone signaling, and their 5´-untranslated region contains a G-rich element that serves as a target sequence for JULGI (Park *et al*., 2025). By targeting a G-rich element in the 5´-UTR, JULGI modulates the translation of *SMXL4/5* mRNA, establishing the JULGI–SMXL4/5 module as an angiosperm-specific regulator of phloem development. Cellular organization innovations are also observed across seed plants; for instance, comparisons between Arabidopsis and *Populus* reveal lineage-specific differences in cambial organization and cell-type specialization. In Arabidopsis, the vascular cambium consists of a single layer of bifacial initials that generate both xylem and phloem mother cells (Smetana *et al*., 2019). In contrast, spatial transcriptomic and high-resolution anatomical studies of *Populus* stems has uncovered a more complex meristem organization, with two distinct meristematic-like cell pools: rectangular procambium-like cells within the phloem domain that generate phloem derivatives, and fusiform cambial-zone cells that produce xylem derivatives (Du *et al*., 2023). This dual organization probably represents an evolutionary innovation that supports differential secondary growth and biomass partitioning between bark and wood tissues.

Traditionally, the fossil record was interpreted as indicating that vascular tissues became more complex, with ancestral forms already possessing primitive xylem whose SCWs progressively elaborated. This view suggested a stepwise acquisition of structural complexity in conducting tissues. However, recent re-interpretations propose that the ancestral vascular system might have consisted of a single type of conducting tissue capable of both water and solute transport (Kenrick and Long, 2026). This perspective aligns with morphological comparisons between the FCCs of bryophytes and the phloem of tracheophytes, which have been proposed to share a common ancestry (Edwards *et al*., 2022). Molecular evidence now provides partial support for this scenario, suggesting that the evolution of conducting tissues involved a mosaic pattern in which deeply conserved regulatory modules coexist with lineage-specific innovations. A comparable example is provided by nitrogen-fixing root nodules, whose evolution is thought to have involved the co-option and rewiring of pre-existing root developmental programs, including components shared with lateral root organogenesis (Schiessl *et al*., 2019). Thus, vascular development provides a representative framework to understand how plant tissue patterning evolved through the interplay between conservation and innovation, integrating molecular and fossil evidence across plant lineages.

## Conclusion

Tracheophytes and bryophytes have evolved distinct but functionally analogous conducting tissues, and emerging molecular evidence suggests that this reflects a combination of ancestral genetic modules, lineage-specific innovations, and rewiring of regulatory networks. In essence, although some independent evolutionary invention has occurred (e.g. pegged rhizoids in certain liverworts), conducting tissues largely represent a mosaic of deep homology, which appears to be the major force underlying their evolution. A full understanding of the evolution of vascular and analogous conducting tissues will require further identification of lineage-specific genes and reconstruction of their regulatory circuit evolution. This will, in turn, provide key insights into how gene regulatory networks have shaped plant tissue patterning over millions of years of evolutionary history.
